# Impact of Virtual Seminars on Hepatitis B Knowledge and Attitudes Among Preclinical-Year Medical Students

**DOI:** 10.7759/cureus.34609

**Published:** 2023-02-03

**Authors:** Kelly Yang, Andrew S Kao, Kaycee Ching, Ronald Thomas, Jocelyn Ang

**Affiliations:** 1 Department of Medicine, Wayne State University School of Medicine, Detroit, USA; 2 Department of Internal Medicine, Wayne State University School of Medicine, Detroit, USA; 3 Department of Family Medicine, Wayne State University School of Medicine, Detroit, USA; 4 Department of Pediatrics, Central Michigan University College of Medicine, Mount Pleasant, USA; 5 Division of Infectious Diseases, Children's Hospital of Michigan, Detroit, USA; 6 Department of Pediatrics, Wayne State University School of Medicine, Detroit, USA

**Keywords:** knowledge, attitudes, medical students, medical education, hepatitis b virus

## Abstract

Background

A limited understanding of hepatitis B virus (HBV) disease transmission contributes to fear of routine contact and can stigmatize affected individuals. To reduce potential HBV-related discrimination, there is a need to increase awareness among medical students on HBV knowledge and transmission. We aimed to assess the impact of virtual education seminars on first- and second-year medical students' understanding of HBV and their attitudes toward HBV infection.

Methods

Pre- and post-seminar surveys were designed and administered to first- and second-year medical students in the February and August 2021 virtual HBV seminars to assess basic knowledge and attitudes toward HBV infection. The seminars consisted of a lecture on HBV followed by case study discussions. Paired samples t-test and McNemar's test for paired proportional differences were used for analysis.

Results

This study included 24 first-year and 16 second-year medical students who completed both pre- and post-seminar surveys. After attending the seminar, participants demonstrated an increase in correct responses to transmission modes including vertical transmission (p≤0.001) and sharing razors or toothbrushes (p=0.031) rather than sharing utensils or shaking hands (p<0.01). Using 5-point Likert means, improved attitudes were observed in concerns of shaking hands or hugging (pre=2.4, post=1.3, p<0.001) and caring for someone with infection (pre=1.55, post=1.18, p=0.009), and acceptance of an HBV-infected coworker in the same workplace (pre = 4.13, post= 4.78, p<0.001).

Conclusion

The virtual education seminars clarify misconceptions about transmission and bias towards individuals with HBV infection. Implementation of educational seminars in medical students’ training is important to improve overall knowledge of HBV infection.

## Introduction

Hepatitis B virus (HBV) infection is a bloodborne disease affecting approximately 257 million lives worldwide, with the risk of subsequent development of cirrhosis and hepatocellular carcinoma accounting for 887,000 deaths per year [1]. In the United States (US), Asian Pacific Islanders (APIs) represent less than 6% of the population but account for at least 60% of HBV infections, exemplifying significant racial disparities for liver cancer compared to the general population [2]. Studies documented that an estimated 50-65% of API patients were unaware of their chronic infection, and less than half had received appropriate treatment [3,4]. Due to its prolonged asymptomatic period, chronic HBV infection may remain undiagnosed for years to decades, and 10-15% of those who develop late-stage complications suffer premature death due to a lack of early recognition and management [5,6].

Although hepatitis B surface antigen has been detected in body fluids including human milk, saliva, and tears, the most potentially infectious sources include blood, serum, semen, vaginal secretions, and also cerebrospinal, synovial, pleural, pericardial, peritoneal, and amniotic fluids [7]. Current literature has shown that many people including medical students frequently mistake transmission modes such as sharing food as the primary cause of spread, leading to erroneous preventive efforts [8,9]. While sexual intercourse and intravenous drug use are the predominant transmission sources in the US, the majority of chronic HBV infections are acquired through vertical transmission [2,10]. Immediate screening is crucial as newborns who acquire HBV perinatally have a nearly 90% risk of developing chronic infection [11,12].

Limited knowledge of HBV transmission contributes to the stigmatization of infected individuals as drug users or sexually promiscuous in need of isolation [13]. The resulting fear of social discrimination and additional sociocultural disparities leads to barriers to timely screening and management [14-16]. Healthcare providers may further stigmatize affected patients as a result of inadequate knowledge of the sociocultural implications associated with chronic HBV infection.

Several cross-sectional studies that examined understanding levels of HBV among medical students have found insufficient knowledge regarding HBV due to a lack of focus in the curriculum [9,17,18]. Case studies are effective interventions for medical students to promote self-directed learning and elicit reflection on their approach to a situation with their knowledge base [19,20]. Continued education plays an important role in demystifying stigmatization and providing proper linkage to care for high-risk populations [21].

Therefore, we conducted a survey study on monthly HBV education seminars offered to medical students at a single academic institution. Using pre- and post-seminar surveys, we aimed to assess the impact of virtual education seminars on first- and second-year medical students' understanding of common HBV transmission routes and attitudes towards those infected with HBV.

## Materials and methods

Study design

Pre- and post-seminar surveys were administered through Qualtrics (SAP, Seattle, WA) in the February 2021 and August 2021 HBV education seminars. Pre-seminar surveys were used to establish a baseline of participants' knowledge and attitudes and to examine how first-year medical students in this study compare to other medical students in other studies. Post-seminar surveys were used to determine the percentage of participants showing improvement following attendance at the seminar. All first- and second-year medical students at Wayne State University School of Medicine (WSUSOM) located in Detroit, Michigan, were invited to attend the seminars via email. A day before the seminar, a Zoom link and pre-seminar survey were emailed to participants who signed up. Participants were emailed the post-seminar survey after the seminar. Participation was voluntary and participants who attended a seminar were given one clinical service learning hour for the Service Learning course that is part of the WSUSOM curriculum. First- and second-year medical students at WSUSOM were included, while participants noting HBV seminar attendance prior to this study were excluded. Incomplete surveys and surveys with no matching pre- and post-seminar six-character alphanumeric codes were also excluded. This study was approved by the Wayne State University Institutional Review Board (IRB-21-05-3588). 

Seminar

The HBV education seminars are an ongoing educational opportunity hosted virtually on the Zoom platform (Zoom Video Communications, San Jose, CA) for preclinical year medical students to increase awareness of HBV transmission and health disparities affecting the API population. The seminars began in 2020 when the pandemic converted many learning opportunities to the online platform. The one-hour seminar consisted of 30 minutes of lecture covering epidemiology, modes of transmission, clinical symptoms, and treatment. The lecture was delivered by a research scientist from the University of Michigan Health System actively involved in HBV prevention and education. The lecture material was adapted from a past presentation on HBV provided by a professor in hepatology at the University of Michigan Health System. After the lecture, participants were randomly assigned to three breakout rooms to discuss three different HBV case studies for ten minutes (Appendix 1). The case studies covered either HBV serology, treatment, and prophylaxis, or medical errors involving delayed prophylaxis and vaccine administration to infants born to mothers with HBV infection [22,23]. Each virtual seminar accommodated a maximum of 30 first-year and 30 second-year medical students. Thus, each breakout had a maximum of 20 participants and one or two facilitators. After 10 minutes, participants reconvened in the general Zoom session for 20 minutes to discuss all of the case studies together; additionally, participants from each breakout room presented their case study.

Survey

Survey questions were derived from previous studies on basic HBV knowledge and attitudes of medical students [17,18]. A pilot study was conducted among 40 medical students during one seminar. This was performed to ensure clarity of questions and to determine the logistics of data collection. The survey took on average under five minutes to complete. The results from the pilot study were not included in the data analysis. The pre-seminar questionnaire consisted of three general sections: a section collecting non-identifiable information from participants (year in medical school, gender, and a non-identifiable six-character alphanumeric code), a section assessing basic HBV knowledge and transmission, and questions assessing attitudes towards HBV and any past learning or personal experiences with HBV. A post-seminar questionnaire was administered immediately after the seminar containing questions similar to the pre-seminar survey and feedback on the seminar. To match individual pre- and post-seminar responses, participants entered the same self-generated six-character alphanumeric code in their pre- and post-seminar responses. Knowledge questions consisted mainly of true and false statements but also included multiple choice or select all that apply questions. Attitude questions were answered using a 5-point Likert scale (1 = strongly disagree, 5 = strongly agree).

Statistical analyses

Paired samples t-test and McNemar’s test for paired proportional differences were used to compare pre- and post-seminar results. A p-value less than 0.05 was considered statistically significant. Statistical analyses were performed using SPSS version 28 (IBM Corp., Armonk, NY).

## Results

A total of 61 students attended the two virtual seminars, including 41 first-year and 20 second year students. In total, 40 medical students completed both pre- and post-seminar surveys, 24 first-year and 16 second-year medical students. Of the total participants, 26 (65%) were male and 14 (35%) were female.

Hepatitis B knowledge

Scores on five of eight true or false statements significantly improved post-seminar (Table 1). Questions one through three assessed knowledge of HBV transmission. After the seminar, significantly more participants understood that HBV is not transmitted by eating food prepared by an infected person (p =0.016) and that HBV can be spread by sharing a toothbrush or razor (p=0.031). Questions four and five evaluated knowledge of chronic HBV infection. A significant increase in participants selecting false to most chronic infections being symptomatic was observed following the seminar (p=0.001). Prophylaxis and vaccine knowledge were evaluated in questions six through eight. Significantly more participants understood the availability of post-exposure prophylaxis after the seminar (p=0.008). Of note, 87.5% correctly answered false to a person never needing the HBV vaccine if he/she has completed the series before. However, after the seminar, fewer students selected false (60%).

**Table 1:** Participant responses to each true or false statement in pre- and post-seminar surveys

	Correct Response, n (%)	
Question Statement (Correct Response)	Pre-seminar	Post-seminar
Hepatitis B can be spread from person to person by eating food prepared by an infected person. (False)	32 (80)	39 (97.5)*
Hepatitis B can be spread from person to person by sharing a toothbrush or razor with an infected person. (True)	33 (82.5)	39 (97.5)*
An infected mother may transmit Hepatitis B to her newborn baby during delivery. (True)	35 (87.5)	38 (95)
Most chronic Hepatitis B infection cases are symptomatic. (False)	27 (67.5)	38 (95)*
People with Hepatitis B can be infected for life. (True)	35 (87.5)	39 (97.5)
Post-exposure prophylaxis is available for Hepatitis B. (True)	28 (70)	36 (90)*
Pregnancy is a contraindication for the use of Hepatitis B vaccine. (False)	27 (67.5)	31 (77.5)
A person never needs Hepatitis B vaccine again if he/she has completed the series before. (False)	35 (87.5)	24 (60)*
*Pre- and post-seminar responses with statistical significance of p<0.05		

A majority of participants before and after the seminar correctly selected sexual transmission and needles as typical routes of transmission (Table 2). While only 45% correctly selected vertical transmission in the pre-seminar survey, this increased significantly to 80% after the seminar (p≤0.001). Before the seminar, 22.5% of participants incorrectly selected both sharing eating utensils or drinking glasses and kissing/handshake as HBV transmission routes. After the seminar, a significant improvement was observed (p<0.01). 

**Table 2:** Pre- and post-seminar responses: How is HBV typically transmitted? (Select all that apply)

Mode of Transmission (Select all that apply)	Pre-seminar, n (%)	Post-seminar, n (%)
Sharing eating utensils or drinking glasses	9 (22.5)	3 (7.5)*
Kissing/handshake	9 (22.5)	1 (2.5)*
Vertical transmission	18 (45)	32 (80)**
Sexual transmission	34 (85)	40 (100)
Needles	35 (87.5)	39 (97.5)
Note: Answer includes vertical transmission, sexual transmission, and needles. *Pre- and post-seminar responses with statistical significance of p<0.01 **Pre- and post-seminar responses with statistical significance of p≤0.001		

Post-seminar survey responses demonstrated a significant increase in awareness that the cultural group with the highest prevalence of hepatitis B is Asians (p≤0.001). In the pre-seminar survey, 45% of the participants knew that Asians were the cultural group with the highest HBV prevalence followed by 22.5%, 20%, and 12.5% selecting Hispanic, Caucasians, and Native Americans, respectively. After the seminar, 100% of the participants selected Asians as the cultural group with the highest HBV prevalence.

Attitudes toward hepatitis B infection

**Figure 1:**
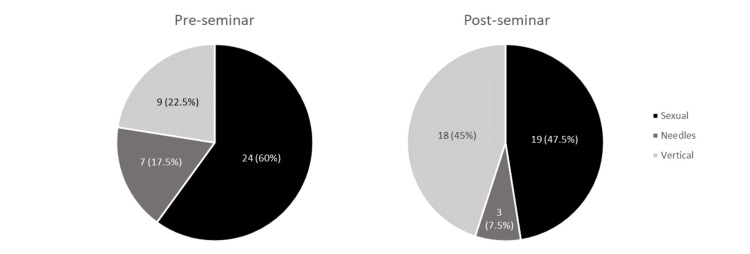
Pre- and post-seminar responses: If a young Asian American woman presents with clinical symptoms of hepatitis B, which of the following is a likely transmission route of her infection?

In the pre-seminar survey, 60% believed sexual transmission to be the likely transmission route in the scenario of a young Asian American woman presenting with hepatitis B, followed by vertical transmission (22.5%) and needles (17.5%). After the seminar, there was a significant increase in choosing vertical transmission (p=0.004). No significant change was observed in participants selecting sexual transmission (p=0.063) and needles (p=0.125) as the likely route of transmission (Figure 1).

A 5-point Likert scale was used to assess medical students’ attitudes toward HBV-infected individuals (Tables 3-4). Following the seminar, a significant increase in mean Likert scale responses was observed in participants accepting an HBV-infected coworker in the same workplace. Significantly more participants strongly disagreed with having concerns about shaking hands or hugging a person infected with HBV or caring for an infected patient on average. No significant change was found in participants believing that all patients should be tested for HBV before they receive health care or feeling uncomfortable talking to someone with HBV infection.

**Table 3:** Pre- and post-seminar survey Likert scale responses to statements assessing attitudes towards HBV

	5-point Likert scale response, n (%)				
Attitude statements	Strongly disagree	Somewhat disagree	Neither agree nor disagree	Somewhat agree	Strongly agree
I believe that all patients should be tested for HBV before they receive healthcare.					
Pre	0 (0)	9 (22.5)	10 (25)	16 (40)	5 (12.5)
Post	1 (2.5)	7 (17.5)	3 (7.5)	18 (45)	11 (27.5)
I would accept an HBV-infected colleague to be in the same workplace or classroom.					
Pre	0 (0)	1 (2.5)	8 (20)	16 (40)	15 (37.5)
Post	0 (0)	1 (2.5)	1 (2.5)	5 (12.5)	33 (82.5)
I have concerns about shaking hands or hugging a person infected with Hepatitis B infection.					
Pre	11 (27.5)	10 (25)	12 (30)	6 (15)	1 (2.5)
Post	34 (85)	3 (7.5)	1 (2.5)	1 (2.5)	1 (2.5)
I would not mind eating from the same plate as a person with Hepatitis B.					
Pre	8 (20)	14 (35)	12 (30)	5 (12.5)	1 (2.5)
Post	3 (7.5)	11 (27.5)	7 (17.5)	7 (17.5)	12 (30)
I would feel uncomfortable having a conversation with someone who has HBV.					
Pre	24 (60)	9 (22.5)	4 (10)	1 (2.5)	2 (5)
Post	35 (87.5)	1 (2.5)	1 (2.5)	0 (0)	3 (7.5)
Caring for a patient with Hepatitis B would make me feel uncomfortable.					
Pre	22 (55)	14 (35)	4 (10)	0 (0)	0 (0)
Post	35 (87.5)	3 (7.5)	2 (5)	0 (0)	0 (0)
Training programs about the occupational risks of bloodborne pathogens, including Hepatitis B, should be offered to all medical students.					
Pre	0 (0)	0 (0)	5 (12.5)	7 (17.5)	28 (70)
Post	0 (0)	0 (0)	2 (5)	3 (7.5)	35 (87.5)
I feel equipped with the skills needed to effectively and safely deal with occupational Hepatitis B risks in healthcare settings.					
Pre	8 (20)	22 (55)	7 (17.5)	2 (5)	1 (2.5)
Post	0 (0)	3 (7.5)	6 (15)	25 (62.5)	6 (15)

**Table 4:** Pre- and post-seminar survey 5-point Likert scale mean responses to statements assessing attitudes towards HBV

	Mean			
Attitude Statements	Pre-seminar	Post-seminar	delta	p-value
I believe that all patients should be tested for Hepatitis B before they receive healthcare.	3.43	3.90	0.47	0.01
I would accept a Hepatitis B-infected colleague to be in the same workplace or classroom.	4.13	4.78	0.65	<0.001
I have concerns about shaking hands or hugging a person infected with Hepatitis B infection.	2.40	1.30	-1.10	<0.001
I would not mind eating from the same plate as a person with Hepatitis B.	2.43	3.38	0.95	0.0024
I would feel uncomfortable having a conversation with someone who has Hepatitis B.	1.70	1.48	-0.22	0.4
Caring for a patient with Hepatitis B would make me feel uncomfortable.	1.55	1.18	-0.37	0.009
Training programs about the occupational risks of bloodborne pathogens, including Hepatitis B, should be offered to all medical students.	4.58	4.83	0.25	0.037
I feel equipped with the skills needed to effectively and safely deal with occupational Hepatitis B risks in healthcare settings.	2.15	3.85	1.70	<0.001

A majority disagreed in the pre-seminar survey that HBV infection control issues have been addressed in the medical school curriculum so far. Moreover, most participants selected that training programs about the occupational risks of bloodborne pathogens should be offered to all medical students in both pre- and post-seminar surveys. After the seminar, a significant increase in mean responses was observed in participants feeling equipped with the skills needed to deal with occupational HBV risks in healthcare settings.

## Discussion

In our study, a significant number of students showed improvement in knowledge of transmission through sharing razors and toothbrushes, rather than through food prepared by an infected person, kissing, handshake, or sharing utensils. Initial survey findings showed that less than half of students (45%) selected vertical transmission as a common transmission route. After the seminar, 80% of participants included vertical transmission as a common route of transmission. All participants demonstrated an understanding that Asian groups are associated with the highest prevalence of HBV infection in comparison to 45% of participants prior to the seminar, and more participants understood the asymptomatic nature of HBV. Prior to the seminar, many participants only partially disagreed with concerns of contracting HBV via shaking hands or hugging someone with active HBV infection, or caring for someone with HBV infection, and partially agreed to accept an HBV-infected coworker in the same workplace. Only after the seminar did most participants endorse strong disagreement and agreement, respectively, to concerns of aforementioned interactions. This demonstrates the effectiveness of seminars in elucidating the misconceptions about transmission and recognizing at-risk populations and the disease nature of HBV. 

Stigmatization may be observed when assessing knowledge of vertical transmission as the most common transmission route in the API population [2]. In our study, a majority of students initially selected sexual transmission as the likely route of transmission in the scenario of a young Asian woman presenting with symptoms of HBV. This exemplifies possible misguided assumptions among medical students toward patients when encountering a case of infectious disease. Cross-sectional studies also reported a low understanding of vertical transmission routes among medical students in Saudi Arabia (51.4%) and India (23.5%) [9,18]. Young people are often associated with higher-risk behaviors of sexual encounters making them more likely to perceive stigma than other groups such as their older counterparts [24]. This may explain the underlying stigma and fear of transmission through close contact. Furthermore, previous studies demonstrated that a majority of medical students perceived that HBV is transmitted by sharing food [9,17,18]. Education of other possible transmission routes could resolve stigmatizing misconceptions directed toward patients with HBV infection.

Unsolicited bias among healthcare workers may serve as an additional barrier to adequate management [24-27]. Judgment among healthcare workers (HCWs) in India included beliefs that transmission resulted from immoral behaviors due to having multiple sexual partners, addiction to drugs, and working in the sex industry [25]. Stigmatization in non-Western culture by healthcare workers has been shown to delay and impair quality care in those with chronic HBV infection [24-27]. They were more likely to exhibit uncaring attitudes toward patients and take additional precautions such as wearing extra gloves to prevent the acquisition of HBV [26,27]. Patients with comorbidities may encounter procedural postponement as most healthcare workers resorted to task-shifting to minimize exposure to viral infection [27]. Psychological repercussions from patients experiencing stigma may render patients less likely to get tested for fear of being diagnosed, causing subsequent social isolation [16]. Patients with greater knowledge of HBV are associated with increased rates of screening [16]. Providers with favorable attitudes toward HBV infection are also associated with improved screening rates [28]. Community-based education intervention is crucial to eliminate inaccurate beliefs such as perceiving HBV as a deadly infection [24,27]. Involving stigmatized patients to share their narratives and targeting multiple levels of stigma, from the individual level to the community level and beyond, should be the focus of future stigma intervention strategies [29].

Contrary to our study results, previous studies reported less than a majority of students in Saudi Arabia (42.1% and 28%) understood that post-exposure prophylaxis is available [17,18]. Interestingly, fewer students after the seminar (60%) compared to 87.5% before the seminar believed patients never needed the HBV vaccine again if they completed the series before. This may be attributed to the seminar’s primary emphasis on disease transmission and associated health disparities. Future seminars should be broadened to highlight revaccination and prophylaxis indications. Similar to the medical students in another study (73.9%), a majority of our participants strongly agreed that training programs on occupational risks of bloodborne pathogens, including HBV, should be provided to medical students [17]. Physicians in training should have basic knowledge of HBV to prevent or detect HBV infection, especially in at-risk populations among their future patients, as early detection and prompt management are crucial to prevent chronic complications.

To our knowledge, this is the first study examining the impact of virtual seminars on medical students’ knowledge and attitudes toward HBV. However, in addition to the small sample size, a limitation of our study was the inability to assess content retention and how attitudes translate into future clinical practice. Additionally, the data was collected from a single institution limiting its generalizability. Given the stigma associated with infectious diseases, social desirability bias needs to be considered and minimized especially in the self-reported attitudes regarding HBV infection. We propose more work to be done examining knowledge retention after the seminar and how the race of survey respondents may affect knowledge and attitude results. Future studies may also include clinical-year medical students to assess how experiences on clinical rotation in the hospital affect hepatitis B knowledge and attitudes.

## Conclusions

A limited understanding of hepatitis B virus (HBV) disease transmission contributes to the stigmatization of affected individuals. Our study demonstrates the use of virtual education seminars in improving knowledge gaps, including misconceptions about common transmission routes, and self-reported attitudes toward HBV infection. Improved understanding of transmission appears to play a role in reducing stigmatizing attitudes toward people infected with HBV. Raising awareness of this stigmatized disease among medical students is a valuable first step in increasing HBV awareness to better prepare them for screening and educating future patients.

## Figures and Tables

**Figure 2:**
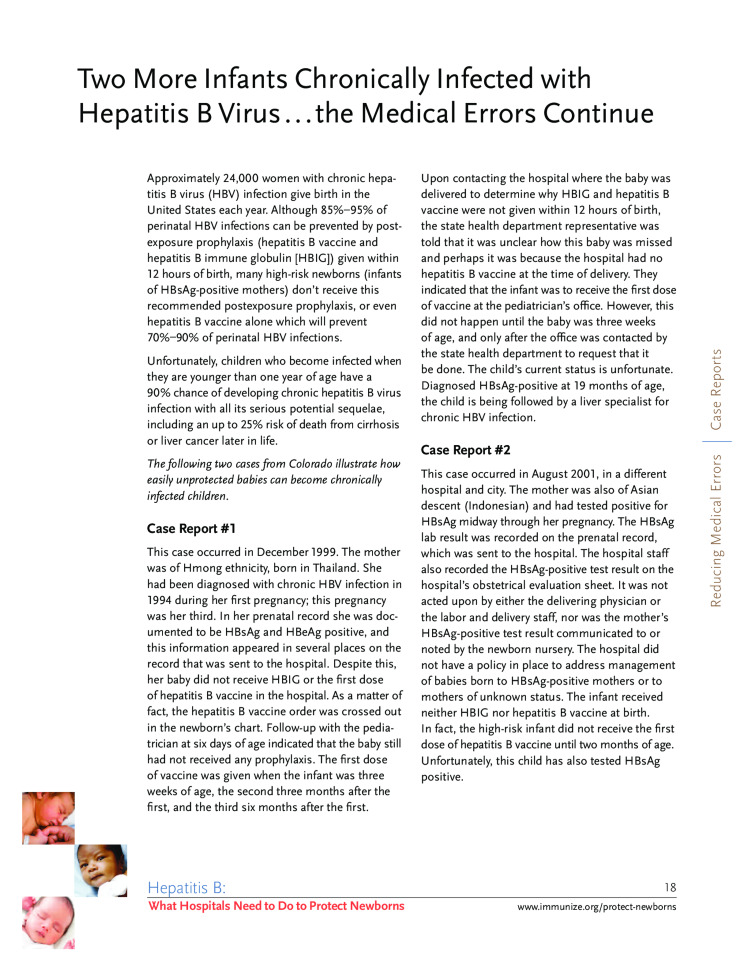
Details of case study 1

**Figure 3:**
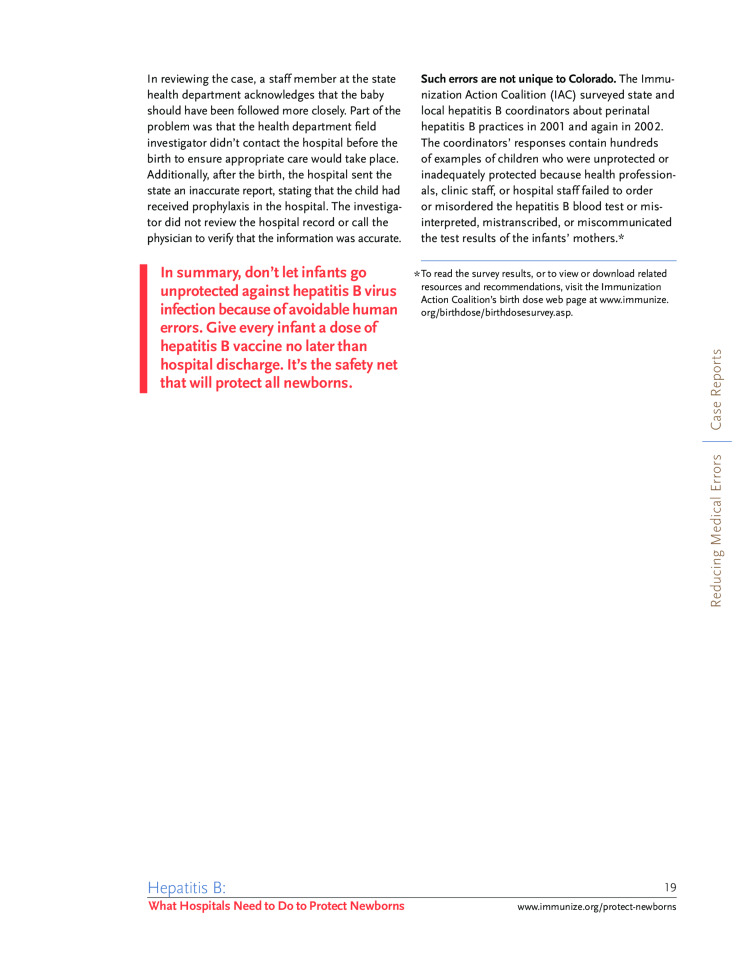
Details of case study 1 continued

**Figure 4:**
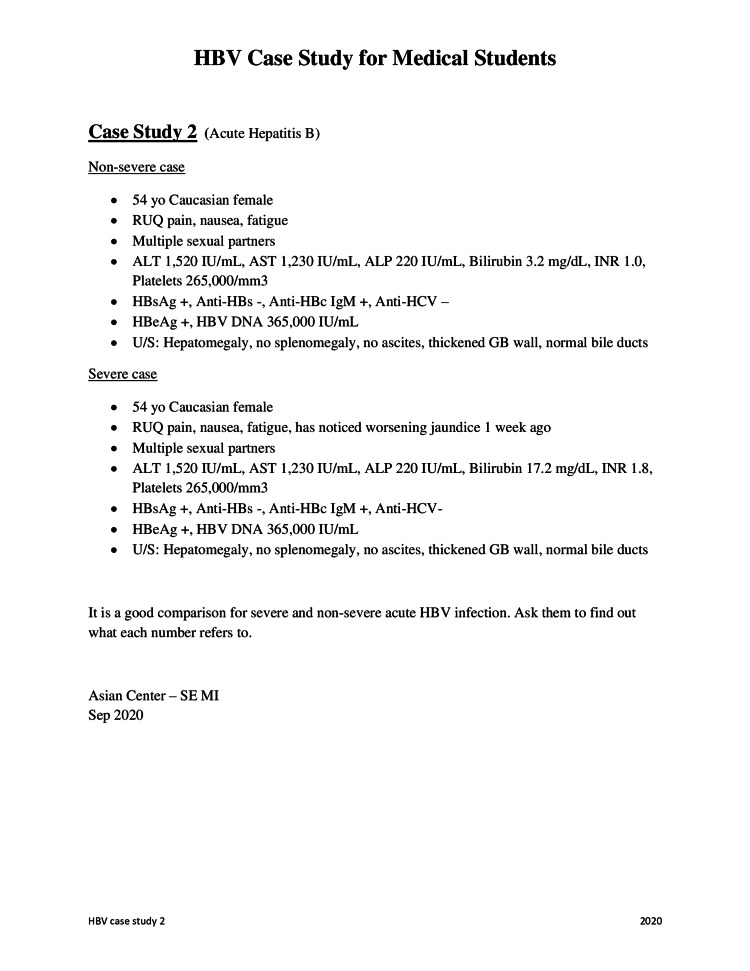
Details of case study 2

**Figure 5:**
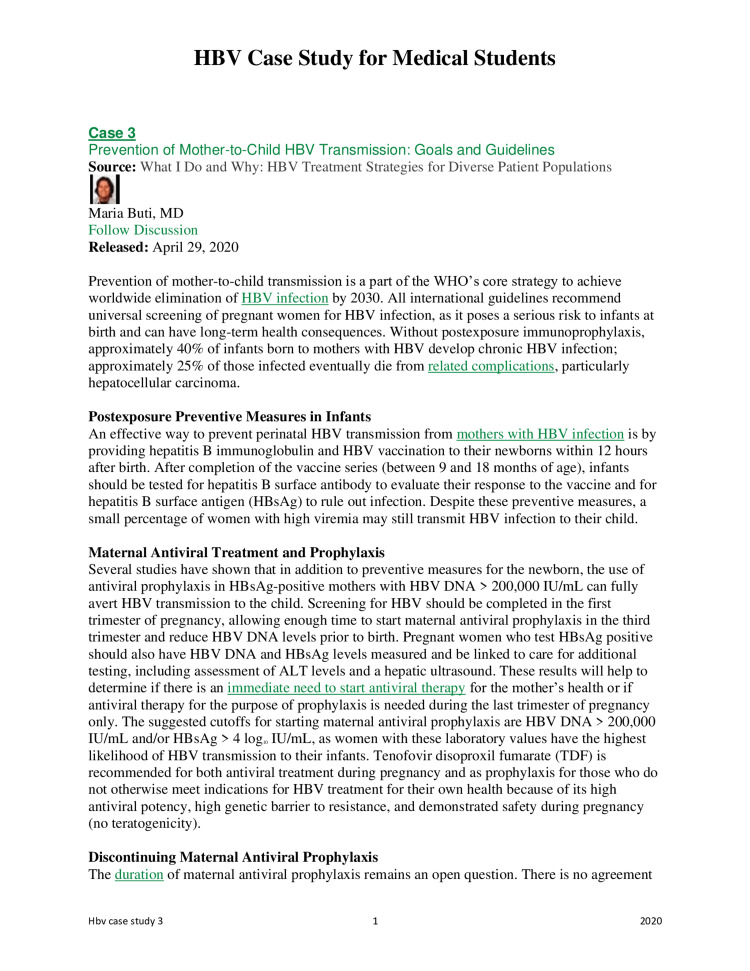
Details of case study 3

**Figure 6:**
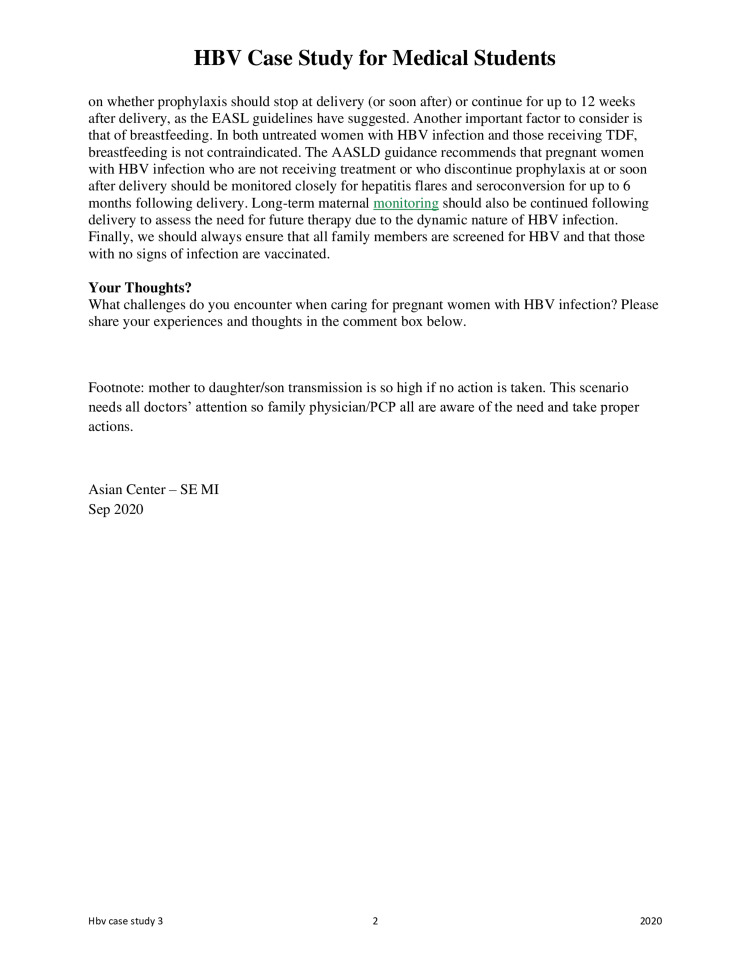
Details of case study 3 continued

## Appendices

The case study in Figures 2-3,  "Two more infants chronically infected with hepatitis b virus...the medical errors continue " by Immunization Action Coalition is licensed under CC BY 4.0. The case studies in Figures 4-6 were given permission for reproduction and use by Asian Center - Southeast Michigan for all educational purposes.
